# Comprehensive quantitation of metabolite concentrations, gross reaction fluxes, and reaction free energies across cells from four organisms

**DOI:** 10.1186/2049-3002-2-S1-P54

**Published:** 2014-05-28

**Authors:** Junyoung Park, Sara Rubin, Yi-Fan Xu, Daniel Amador-Noguez, Jing Fan, Joshua Rabinowitz

**Affiliations:** 1 Lewis-Sigler Institute for Integrative Genomics, Princeton University, Princeton, NJ, USA; 2 Department of Chemical and Biological Engineering, Princeton University, Princeton, NJ, USA; 3 Department of Chemistry, Princeton University, Princeton, NJ, USA

## Background

Current technological limitations hinder the measurement of certain core metabolites, including glyceraldehyde-3-phosphate and erythrose-4-phsophate. For measurable metabolites, absolute concentration data sets are not always thermodynamically consistent. The use of a previously underappreciated thermodynamic relationship, however, provides a way to remedy these shortcomings. We develop a novel approach to integrate metabolite concentrations, reaction fluxes, and reaction free energies (Δ_r_G) and apply it across four organisms: *E. coli*, *C. acetobutylicum*, Baker’s yeast, and mouse renal epithelial cells.

## Materials and methods

Microbes were grown in 1,2-13C, 3-13C, U-13C, and 50% U-13C glucose minimal media. Mouse renal epithelial cells were cultured in 1,2-13C glucose, U-13C glucose, and U-13C glutamine Dulbecco’s Modified Eagle Media, supplemented with 10% dialyzed fetal bovine serum. We measured intracellular isotope distributions via LC/MS and calculated absolute metabolite concentrations using an isotope-ratio-based approach [1]. Nutrient uptake and metabolite excretion rates were determined by 1H-NMR. Standard reaction free energies (Δ_r_G°) were computed using component contribution [2] at physiological pH and ionic strength.

## Results

Our approach was applied to yield *in vivo* Δ_r_G values and metabolite concentrations in *E. coli*, *C. acetobutylicum*, Baker’s yeast, and mouse renal epithelial cells. For each organism, free energies for many reactions in central carbon, biosynthesis, and folate metabolism were determined from ratios of forward to reverse fluxes. We combined these values with concentration measurements for ~100 metabolites and Δ_r_Gº values to determine global core metabolite concentrations more completely and precisely. In general, amino acids comprise a large fraction of the total metabolite pool. Energy charge was higher in mammalian and *E. coli* cells whose main route of ATP generation is through oxidative phosphorylation. Certain reversible reactions of glycolysis (fructose-bisphosphate aldolase and triose-phosphate isomerase) were also more strongly forward driven in the oxidative cell types, especially in the mammalian cells, perhaps reflecting minimal need to maintain equilibrium at these steps when ample free energy is available via other routes.

## Conclusions

Integrative analysis of metabolite concentrations with reaction fluxes and free energies should enable substantially more comprehensive and precise characterization of cellular metabolic activity.
